# Structure, Evolution and Functional Inference on the CDP Gene Family in European Hazelnut

**DOI:** 10.3390/ijms27198924

**Published:** 2026-10-08

**Authors:** Ciro Gianmaria Amoroso, Barbara Auzino, Adriana Rita Evangelista, Rosa Pilolli, Chiara Nitride, Giuseppe Andolfo

**Affiliations:** 1 Department of Agricultural Sciences, University of Naples Federico II, Via Università 100, 80055 Portici, Italy; 2 Institute of Sciences of Food Production, National Research Council (ISPA-CNR), Via Giovanni Amendola 122/O, 70126 Bari, Italy

**Keywords:** *Corylus avellana*, food allergens, functional inference, proteomic, promoter signatures

## Abstract

Food allergy is an IgE-mediated response of the immune system of sensitive subjects to specific proteins. The recent WHO/FAO consultation included hazelnut (*Corylus avellana* L.) in the list of global priority allergenic ingredients. One of the most common hazelnut allergen groups encompasses the Cupin_1 domain-containing proteins (CDPs). To date, three CDP allergens (*Cor a 9*, *Cor a 11*, and *Cor a 16*) have been deposited into the official WHO/IUIS database. Recently, using a whole-genome approach, the *Cor a* loci were mapped in four *Corylus* species. However, the task of identifying novel CDP allergen candidates directly within the genome has not yet been addressed. The recently available *C. avellana* cv. ‘Tombul’ genome sequence allowed the identification of 114 hazelnut CDP-encoding genes. The maximum likelihood analysis allowed us to reconstruct the evolutionary relationships underlying the diversification and expansion of the CDP superfamily. Several duplication events associated with lineage-specific evolution were discovered. Interestingly, conserved epitopes and amino acid motifs that likely play a role in allergenic function were also identified. A total of 42 cis-acting regulatory elements were shared in the promoter regions of known and new putative allergens. Using the well-known CDP allergens as an internal benchmark, we have identified and molecularly analyzed new CDP allergen candidates. To provide a first transcriptional characterization, *Cor a 9*, *Cor a 11*, and *Cor a 16* were analyzed by real-time PCR. The expression analysis showed generally low relative transcript levels of Cor a genes in immature reproductive tissues compared with young leaves. Interestingly, mass spectrometry analysis confirmed the accumulation of known allergens and several predicted CDP allergen candidates in commercial hazelnut flour. The data reported here may help to further address food safety questions and could be conveniently used in breeding research aimed at developing hypoallergenic cultivars.

## 1. Introduction

Ranked among the most nutritious nuts in the world [1], hazelnuts (*Corylus avellana* L.) belong to the *Betulaceae* family. Hazelnuts have been an integral part of the human diet for millennia. Archaeological evidence and historical records confirm that hazelnuts were consumed as early as 9000 years ago and have maintained a significant presence in the diets of diverse cultures, particularly in Mediterranean regions [2]. However, recent market analyses revealed an expansion in global hazelnut demand, driven both by an increasing awareness of their nutritional properties and their widespread use in the confectionery industry [3]. Despite its extensive use, *C. avellana* is also recognized as a significant source of IgE-mediated food allergies, inducing symptoms ranging from simple urticaria and oral allergic syndrome to extremely harmful, even life-threatening reactions [4]. Sometimes, even small traces of hazelnut protein can cause severe reactions in sensitive individuals. Therefore, precautionary allergen labelling practices are necessary to reduce accidental exposure due to cross-contamination during food processing [5].

The allergenic profile of hazelnuts is complex and comprises highly diverse proteins belonging to distinct families. Among these, the Cupin_1 domain-containing protein (CDP) superfamily is predominantly composed of seed storage proteins (SSPs) that are evolutionarily conserved and play a dual role in plant physiology and allergenicity [6]. The CDP superfamily includes proteins belonging to germins (<7S proteins), vicilins (7S globulins), and legumins (11S globulins) groups [7]. Within this family, the 11S legumin *Cor a 9* is the best-characterized allergen, and along with the 7S vicilin *Cor a 11*, is strongly linked with allergenic reactions, including anaphylaxis, especially in children and individuals with primary hazelnut allergy [8]. Recent genomic analysis of *C. avellana* identified a new allergen, *Cor a 16*, which is the N-terminal fragment of 7S globulin located on chromosome four. Its high level of immunoreactivity underscores the clinical significance of this discovery; in recent testing, *Cor a 16* was found to be reactive in 8 out of 11 sera from allergic subjects, marking it as a critical target for understanding hazelnut hypersensitivity [9]. In this context, the availability of high-quality chromosome-scale genome assemblies for *Corylus* spp. allowed the dissection of allergenic gene families at an unprecedented level of detail and facilitated the discovery of other potentially allergenic proteins [8,10]. The availability of bioinformatic tools designed for allergen prediction and epitope analysis could promote the discovery of new potential allergenic genes directly in the plant protein families [11,12,13]. To date, a whole-genome analysis to identify novel CDP allergen candidates in a crop has not yet been undertaken, although the CDP superfamily has been well-characterized in many plant species [14,15,16,17,18]. Therefore, in this study, we provide the first genome-wide characterization of the CDP superfamily in *C. avellana*, identifying 114 genes and analyzing their structural and evolutionary features. Furthermore, for the first time, we applied a combined approach using multiple bioinformatic tools to predict both linear and conformational epitopes and to estimate overall protein allergenicity, leading to the identification of new CDP allergen candidates in *C. avellana.* Gene expression analysis was performed on immature reproductive tissues of *C. avellana* cv. Mortarella, whereas protein accumulation was investigated in commercial hazelnut flour. Finally, we investigated CDPs’ cis-acting regulatory elements (CREs), focusing on the newly identified allergen candidates and providing insights into potential transcriptional control mechanisms associated with their expression. Moreover, mass-spectrometry confirmed the accumulation of well-known CDP allergens and novel candidates. Together, these data provide a prioritized list of high-confidence allergen candidates that can guide future gene-editing experiments aiming at developing hypoallergenic cultivars.

## 2. Results

### 2.1. Genomic Characterization of Cupin_1 Domain-Containing Protein Encoding Genes

To date, only three hazelnut CDP (*Cor a 9*, *Cor a 11* and *Cor a 16*) sequences have been deposited into the official allergen database (www.allergen.org, accessed on 15 July 2025). The task of identifying CDP-encoding genes directly within the hazelnut genome has not yet been addressed. Using a hybrid gene annotation strategy, 114 CDPs (Supplementary Table S6), including three known Cor a allergens, were identified in the hazelnut genome. The CDP superfamily included 99 monocupins and 15 bicupins, which have one and two Cupin_1 domains, respectively (Supplementary Table S7). CDP-encoding sequences had a gene length ranging from 622 bp to 9825 bp, with exon numbers ranging from 1 to 16 (Supplementary Table S6). The average protein length was 271 amino acids, whereas the average length of the Cupin_1 domain was 164 amino acids (Supplementary Table S6).

**Figure 1:**
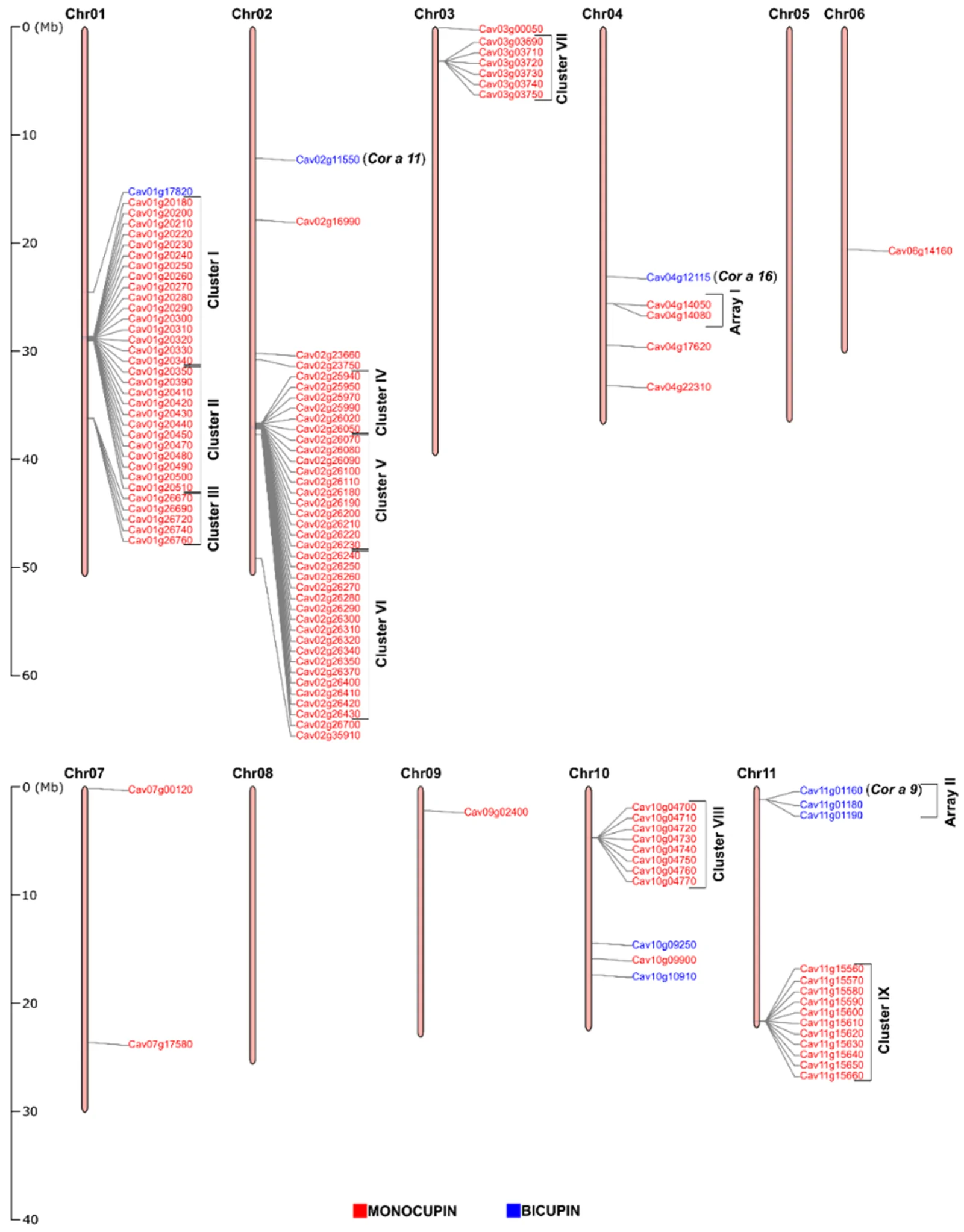
Physical map of hazelnut (*C. avellana* cv. Tombul) CDP-encoding gene clusters. Hazelnut chromosomes are represented as pink bars. The approximate location of each CDP-encoding gene cluster and tandem array is marked and numbered (I–IX). CDP-encoding genes, either in a cluster or in a tandem array, are indicated with a gene ID. The colour used for each ID indicates the structural class to which the encoded protein (monocupin and bicupin) belongs.

Genomic distribution of CDP loci was not random (X^2^ = 109.1; *p* < 0.01) (Figure 1). Indeed, no CDP-encoding genes were present on chromosomes 5 and 8. 107 CDP-encoding genes were on chromosomes 1, 2, 3, 10, and 11, and only seven loci were found on chromosomes 4, 6, and 9 (Figure 1). About 64% (73 out of 114) of CDP-encoding genes were on chromosomes 1 and 2, which were grouped in clusters I–VI (Supplementary Table S8). Clusters on chromosome 2 encompassed 33 monocupin encoding genes (Figure 1). Six CDPs were found in Cluster VII on chromosome 3. Two arrays (AI and AII), including two and three genes, were identified on chromosomes 4 and 11, respectively. Finally, two clusters, Cluster VIII (8 genes) and Cluster IX (11 genes), were on chromosomes 10 and 11, respectively (Figure 1).

### 2.2. Allergenic Potential Estimation of CDP-Encoding Genes

To discover novel allergen candidates in the hazelnut CDP superfamily, we performed a combined analysis using three allergen predictors, based on structure similarity, statistical, machine learning, and deep learning methods. The 114 CDPs were analyzed using AlgPred [11], which returned hybrid scores ranging from 0.48 to 1.98. A higher hybrid score generally indicates a stronger prediction of allergenicity. To complement these results, we performed a second prediction using AllergyPred [13]. AllergyPred’s probability score ranges from 0 to 1. Only 15 CDPs (out of 114 CDPs) had a probability score > 0.7 and were predicted as allergens (Supplementary Table S9). Furthermore, to assess the allergenic potential of CDPs, we estimated the identity score of 3D surface epitopes using AllerCatPro [12]. A 3D surface epitope similarity score > 93% is classified as ‘strong evidence’. Overall, the identity score ranged from 31.6% to 100%, and a total of 1357 conformational epitopes were identified by AllerCatPro (Supplementary Table S9).

Subsequently, to prioritize CDPs with high allergenic potential, we applied an AllergyPred probability score ≥ 0.98 together with an AlgPred hybrid score ≥ 1, which allowed the identification of eight CDPs (Table 1). This subset included *Cor a 9*, *Cor a 11*, *Cor a 16*, and five other CDPs: two on chromosome 4 (Cav04g14050 and Cav04g14080) and three on chromosome 11 (Cav11g01180, Cav11g01190, and Cav11g15560) (Table 1). Cav11g01180 and Cav11g01190 were previously classified as *Cor a 9* isoallergens [10]. AllerCatPro was used as complementary structural evidence and was not included among the selection criteria. Five of the eight prioritized CDPs showed a 3D epitope identity > 93%, corresponding to strong evidence according to AllerCatPro, while *Cor a 16* showed 92.9% and Cav04g14050 and Cav04g14080 showed 86.7%.

**Figure 2:**
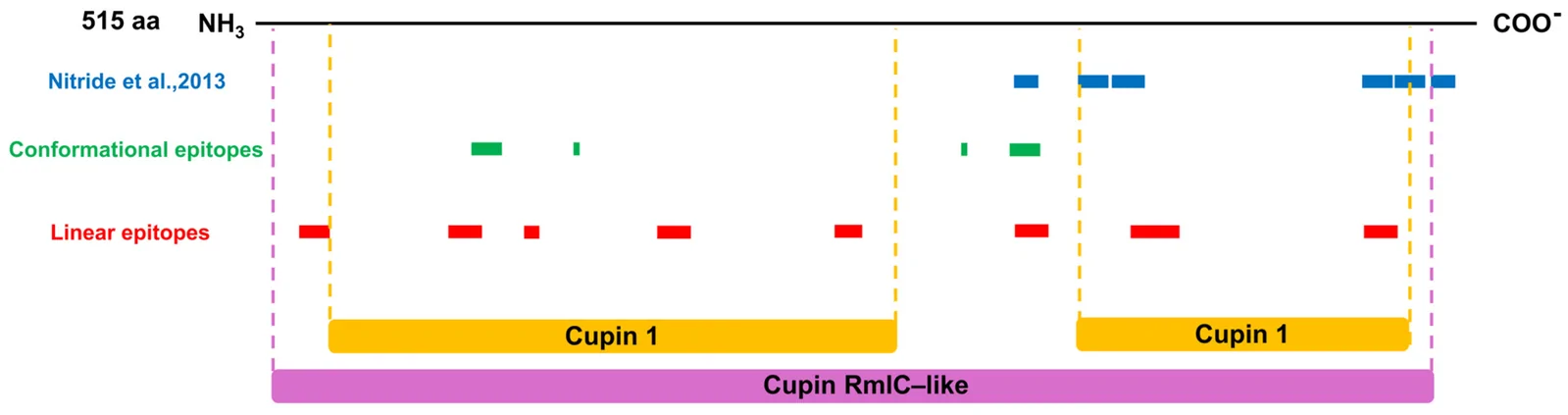
Schematic representation of the allergenic protein *Cor a 9* (Cav11g01160.1) from *C. avellana*. The two Cupin 1 domains (pfam00190) in yellow are highlighted within the broader Cupin_RmlC-like region (PLN00212) in purple. Oligopeptides reported by Nitride et al. [19] are shown in blue, while conformational and linear epitopes are indicated in green and red, respectively.

AllergyPred identified 71 linear epitope occurrences across the eight prioritized CDPs, while AlgPred predicted several additional linear epitopes, particularly in *Cor a 9* and its isoallergens (Supplementary Table S9). Interestingly, the SLL epitope was identified by AllergyPred in six of the eight prioritized CDPs. For *Cor a 16* (Cav04g12115), AllergyPred and AlgPred identified two common linear epitopes (QEEEGG and RNFLA), whereas no linear epitope was predicted by AlgPred for *Cor a 11*. *Cor a 9* showed the highest number of predicted linear epitopes and shared six AllergyPred-predicted epitopes with its isoallergens (Cav11g01180 and Cav11g01190). In addition, 27 conformational epitopes were predicted for *Cor a 9* by AllerCatPro (Supplementary Table S9). Nitride et al. [19] identified six immunoreactive oligopeptides, of which five were mapped within the second Cupin_1 domain of *Cor a 9*, where several of the predicted linear and conformational epitopes are concentrated (Figure 2). In particular, the peptide GFEETICSLR (residues 321–330) maps to a region of overlap between both epitope types, indicating a potential IgE-binding hotspot.

### 2.3. Reconstructing Evolutionary Relationships of Cdp-Encoding Genes

**Figure 3:**
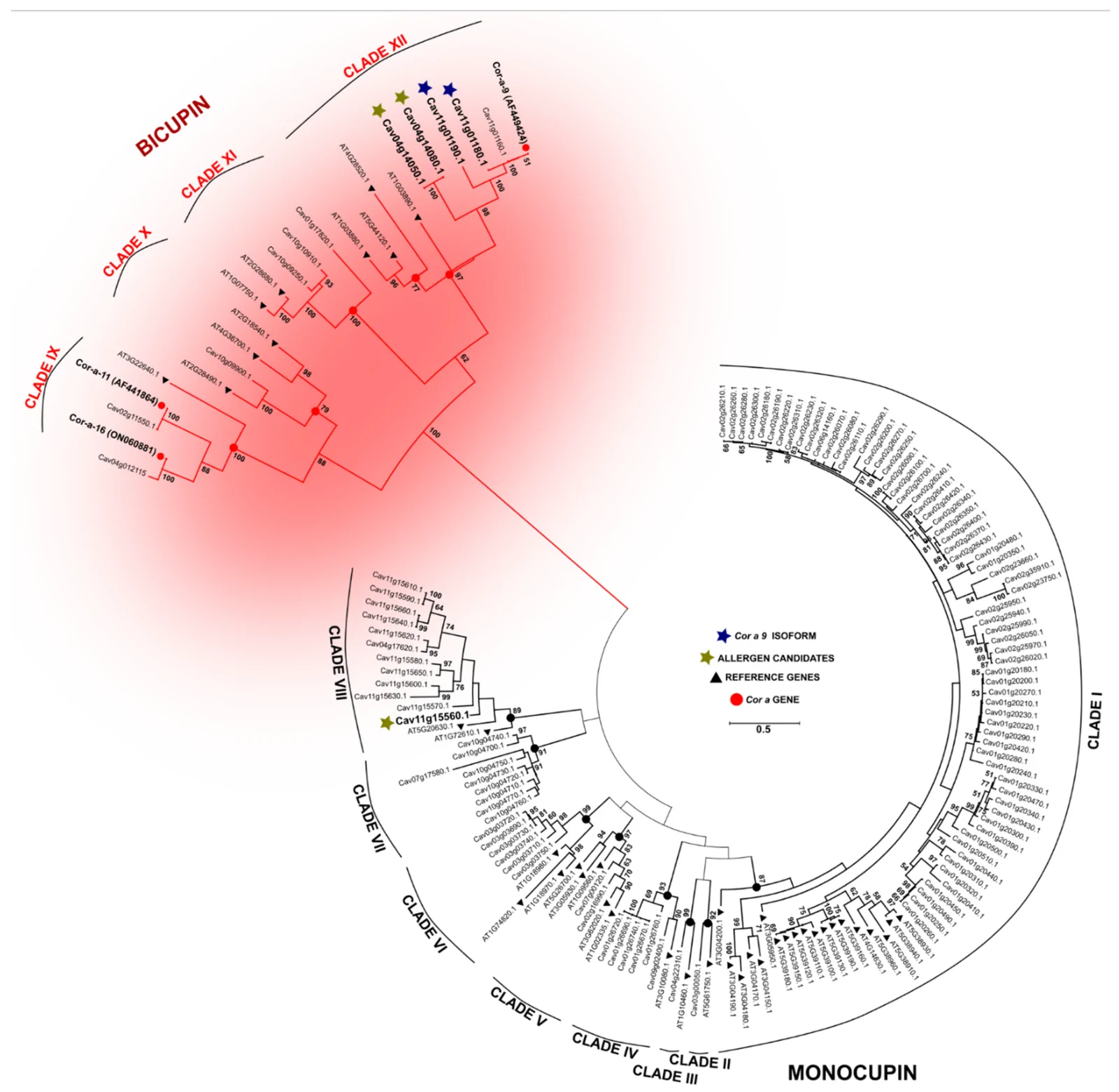
Phylogenetic analysis of the hazelnut CDP superfamily. A phylogenetic tree was inferred using the maximum likelihood method based on the JTT model. The bicupin superclade is drawn with a red background. Twelve clades (I–XII) are indicated in the tree. The tree is drawn to scale, with branch lengths measured in the number of substitutions per site. The black triangle, red spot, and gold star mark *Arabidopsis thaliana* CDPs, hazelnut allergens, and new allergen candidates, respectively; the blue star marks the *Cor a 9* isoforms.

To retrace the evolutionary history of the hazelnut CDP superfamily, we performed a maximum likelihood analysis. The phylogenetic analysis included 114 *C. avellana* CDPs, including three known hazelnut allergens (*Cor a 9*, *Cor a 11*, and *Cor a 16*). Additionally, 42 CDPs identified by He et al. [16] in *A. thaliana* were included as a comparative reference to support the evolutionary and functional interpretation of the hazelnut CDP family. Hazelnut CDPs were collapsed into two robust superclades (monocupins in black and bicupins in red) supported by a bootstrap value of 100 (Figure 3).

The superclades were further divided into 12 clades, denoted by Roman numerals (I–XII) (Figure 3). Clade I grouped 66 and 19 CDPs of hazelnut and *A. thaliana*, respectively. Interestingly, hazelnut CDPs showed ~87% protein sequence homology. In contrast, clades II and III were the smallest, with only one hazelnut sequence. Moreover, clades IV and V included six and two hazelnut CDPs, respectively. No *A. thaliana* CDP homolog was identified in clade VII, which consisted entirely of *C. avellana* sequences. Six and twelve hazelnut CDPs clustered in clades VI and VIII, respectively (Figure 3).

Notably, *Cor a 16* (Cav04g12115), *Cor a 11* (Cav02g11550), *Cor a 9* (Cav11g01160), and its isoforms Cav11g01180 and Cav11g01190 were part of the bicupin superclade, which was divided into four clades (IX–XII). Clade IX included *Cor a 16* and *Cor a 11* together with the *A. thaliana* CDP AT3G22640.1. Clade X contained Cav10g09900 as the only hazelnut CDP and included three *A. thaliana* CDPs (AT2G18540.1, AT2G28490.1, and AT4G36700.1); Cav10g09900 showed ~50% sequence homology to AT2G28490.1, a vicilin-like seed storage protein [20]. Three hazelnut and two *A. thaliana* CDPs clustered in clade XI. Finally, clade XII included *Cor a 9*, its isoforms Cav11g01180 and Cav11g01190, and the candidate CDPs Cav04g14050 and Cav04g14080, together with four *A. thaliana* CDPs (AT1G03880.1, AT1G03890.1, AT4G28520.1, and AT5G44120.1). Interestingly, CDP-encoding genes clustered within the same phylogenetic clade were preferentially organized in genomic clusters. These genes most likely originated from recent duplication events [21]. A total of 38 tandem duplication pairs, involving 60 unique CDP-encoding genes, were identified (Supplementary Table S10).

To discover enriched and/or conserved amino acid motifs associated with allergenicity, we performed a MEME D-mode analysis on CDPs. No over-represented motifs were identified in *Cor a 9*, *Cor a 11*, *Cor a 16*, and the new allergen candidates compared to the remaining CDPs. Two conserved motifs (M1: PAG and M2: GDI) were identified in Cor a allergens and were also reported by the epitope predictors.

### 2.4. Preliminary Molecular Analysis and Cis-Regulatory Element (CRE) Prediction

To monitor transcriptional changes in three hazelnut allergens (*Cor a 9*, *Cor a 11*, and *Cor a 16*), qRT-PCR analyses were performed on unripe seed tissues. Globally, low relative expression levels were detected for the three Cor a genes in the analysed reproductive tissues compared with young leaves (Supplementary Figure S3). *Cor a 9* and *Cor a 16* showed lower relative expression in all analysed tissues, whereas *Cor a 11* showed higher relative expression in bracts than in the other reproductive tissues. Therefore, these findings led us to investigate the putative promoter regions (PPRs) of CDPs and to characterize their cis-regulatory elements (CREs). A total of 54,970 CREs were predicted in the PPRs of CDP-encoding genes (Supplementary Table S4), which were grouped in six functional categories, described by Andolfo et al. [22]. A common subset of 42 CREs was identified in the PPRs of the novel allergen candidates (Table 1), which was also present in the three Cor a PPRs (Supplementary Table S5; Supplementary Figure S2). Two CREs (ABREMOTIFAOSOSEM and ACGTABREMOTIFAOSOSEM) were significantly enriched (FDR < 0.05) in the PPRs of the three Cor a genes and novel candidate allergens (Table 1). These elements were strong classifiers of ABA up-regulated promoters, and also significantly enriched in classes of genes known to be regulated by ABA [23]. Among these, the most represented CREs belonged to the TSA category. In particular, CACTFTPPCA1, EBOXBNNAPA, GTGANTG10, POLLEN1LELAT52, and ROOTMOTIFTAPOX1 represented ~35% (712 out of 2113) of the total identified CREs. Comparison of the normalized CRE occurrences in the PPRs of allergen candidates revealed a common profile (Duncan’s Multiple Range Test). Interestingly, tissue-specific CREs showed a significant difference compared to the remaining classes. Similarly, both hormone and metabolic CREs showed a significant difference (Table 2).

### 2.5. CDP Allergen Detection in Hazelnut Mature Edible Kernel

To investigate the accumulation of known allergens and predicted CDP allergen candidates, proteins extracted from commercial hazelnut flour were analyzed by LC–MS/MS. Mass spectrometry confirmed the presence of *Cor a 9* (Cav11g01160) and its isoforms (Cav11g01180 and Cav11g01190) (Table 3). In addition, peptides corresponding to Cav04g14050 and Cav04g14080 and *Cor a 11* (Cav02g11550) were detected, providing proteomic evidence for the accumulation of known allergens and predicted CDP allergen candidates in mature hazelnut kernels. Importantly, three peptides (YLQLSAER, GVEETFCTLR, and AIPEDVLINSYR), mapped to the immunoreactive 20.7 kDa polypeptide by Nitride et al. [19], were unambiguously assigned to two novel CDP allergen candidates (Cav04g14050 and Cav04g14080) by manual inspection (Table 3). The assignment of these immunoreactive polypeptides to the predicted CDP allergen candidates provides additional evidence supporting their potential immunoreactivity. Mass spectrometry analysis enabled the identification of 53 unique peptides exclusively assigned to *Cor a 16* (Cav04g12115) (Table 3), including the de novo sequence YTRDPQQQYQQ [9]. Furthermore, a total of 22 peptides were specifically assigned to Cav10g09900, which was predicted as an allergen by AllergyPred but showed probability and hybrid scores lower than our imposed cut-off (Supplementary Table S9). Finally, no peptides were mapped to Cav11g15560, which may not have been expressed in the mature kernel (Table 1).

## 3. Discussion

The Cupins constitute a functionally diverse protein superfamily, which together with the prolamin superfamily exemplify class I food allergens [24,25]. These proteins, characterized by a conserved β-barrel domain and two conserved motifs, are highly stable to thermal denaturation, acidic environment, and gastrointestinal proteases and can cause moderate to severe allergic reactions [26]. So far, 13 hazelnut allergens have been found in *C. avellana* L. [10], of which *Cor a 9*, *Cor a 11*, and *Cor a 16* are CDPs. The identification and functional validation of the hazelnut CDPs may hold promise for breeding improved crops with hypoallergenic traits, using modern biotech approaches [27,28]. Previous genome-wide studies have characterized Cupin-related gene families in several plant species [14,16,18]. However, the comprehensive characterization of the CDP superfamily in hazelnut remains largely unexplored. Leveraging the high-quality genome assembly (CavTom2PMs-1.0) of *C. avellana* [8], this study provides the first comprehensive genome-wide identification and characterization of the CDP superfamily in this economically important crop. Our genome-wide analysis identified 114 CDP loci, compared with 42 CDPs identified in *A. thaliana* [16]. Together with the extensive tandem duplication observed in hazelnut, this is consistent with a lineage-specific expansion of the CDP family during hazelnut evolution. The distribution of CDP-encoding genes in the hazelnut genome was highly uneven, with ~84% organized in gene clusters or arrays. A total of 60 CDP-encoding genes formed 38 tandem duplication pairs, supporting a major contribution of tandem duplication to CDP family expansion. Gene duplication is a primary driver for gene family expansion, enabling plants to diversify their genetic repertoire, thereby facilitating species differentiation and enhancing environmental adaptability [21,29].

CDPs can exert a variety of physiological functions [30], such as providing nutrients for plant growth and development, participating in enzymatic reactions, acting as transcription factor cofactors and redox sensors in cell signalling pathways, and contributing to plant defence responses [31,32]. The functional diversity of this gene superfamily is also reflected in the structural variability of hazelnut CDP loci, as shown by differences in gene length (from 622 bp to 9825 bp) and exon number (from one to sixteen). Gene structural diversity has been proposed as an important mechanism in the evolution of multigene families [33], and significant differences in gene structure among CDP families have also been reported [18]. The maximum likelihood analysis grouped hazelnut CDPs into 12 clades (I–XII) and two major superclades, corresponding predominantly to monocupins and bicupins. The bicupin superclade included all the known CDP allergens, *Cor a 9*, *Cor a 11*, and *Cor a 16*. Although predominantly composed of bicupins, this superclade also included some monocupin sequences showing a closer evolutionary relationship with bicupins than with the remaining monocupins (Figure 3). Dunwell et al. [34] proposed that bicupins evolved through duplication and subsequent fusion of a single-domain ancestor. Our phylogeny suggests that bicupins might also have lost a Cupin domain during evolution, generating monocupins evolutionarily related to bicupins. A similar diversification pattern was also proposed by He et al. [16] in *Brassica napus*. Beyond the evolutionary comparison, *A. thaliana* also provided a useful functional reference for hazelnut CDPs. Among the functionally characterized Arabidopsis CDPs, some examples were particularly relevant because they clustered with hazelnut allergens or allergen candidates. In clade XII, *Cor a 9*, its isoforms Cav11g01180 and Cav11g01190, and the candidate CDPs Cav04g14050 and Cav04g14080 clustered with the Arabidopsis cruciferins CRUA, CRUB, and CRUC. A triple-knockout study of CRUA, CRUB, and CRUC showed that mutant seeds were more sensitive to artificial ageing and exhibited increased protein oxidation [35]. In clade IX, *Cor a 11* and *Cor a 16* clustered with the vicilin-like protein PAP85, whose knockdown and rescue experiments showed its involvement in TMV accumulation and infection-associated endoplasmic reticulum remodeling [36]. Similarly, Cav10g09900 clustered with VL22, whose C-terminal region was shown to mediate vacuolar targeting [37]. These phylogenetic relationships suggest that the corresponding hazelnut CDPs may have similar biological roles.

A total of eight CDPs, including the three well-known allergens, two *Cor a 9* isoforms (Cav11g01180 and Cav11g01190), and three newly identified candidates (Cav04g14050, Cav04g14080, and Cav11g15560), showed high allergenicity scores. These eight CDPs were prioritized based on a hybrid score ≥ 1.0 and an AllergyPred probability score ≥ 0.98, while AllerCatPro provided complementary information, with 3D epitope identity values > 86% for all eight prioritized CDPs. Overall, AllerCatPro identified 1357 conformational epitopes across the CDP dataset, while AllergyPred and AlgPred predicted several linear epitopes in the prioritized subset (Supplementary Table S9). Notably, the predicted SLL epitope was present in six of the eight prioritized CDPs. Furthermore, the identification of conserved motifs such as PAG and GDI among both known allergens and novel candidates could suggest a recurrent immunogenic pattern within hazelnut CDPs. The density of both linear and conformational epitopes in *Cor a 9* was particularly interesting. This concordance between in silico predictions and the known allergenic properties of *Cor a 9* supports the use of these analyses for prioritizing potential IgE-reactive CDPs and identifies potential IgE recognition sites. Altogether, these results indicate that bicupin-type proteins represent the major allergenic group within the hazelnut CDP family [6,7]. In this study, *Cor a 9*, *Cor a 11*, and *Cor a 16* generally showed low relative transcript levels in immature reproductive tissues compared with young leaves. This pattern may be related to the developmental stage of the fruit, since SSPs typically accumulate during later stages of seed maturation [8,38]. Indeed, Garino et al. [39] reported higher *Cor a 11* transcript levels in ripe than in unripe seeds. In addition, *Cor a 11* transcript levels varied among hazelnut cultivars and across different stages of fruit development [39]. To our knowledge, transcriptional profiles of *Cor a 9* and *Cor a 16* in hazelnut tissues have not been reported.

Therefore, to explore potential regulatory features associated with CDP expression, we examined their promoter regions, focusing on CREs associated with developmental, hormonal, and stress-responsive regulation. In particular, the detected CREs were grouped into six functional categories as previously described by Andolfo et al. [22]. Among these, 42 CREs were consistently shared by all high-confidence allergen candidates and the known Cor a genes, including the ABA-responsive motifs ABREMOTIFAOSOSEM and ACGTABREMOTIFAOSOSEM [40]. The presence of these elements suggests a possible association with ABA-responsive regulation during seed maturation or under specific stress conditions [41]. In addition, the high frequency of elements such as CACTFTPPCA1, GTGANTG10, POLLEN1LELAT52, and ROOTMOTIFTAPOX1 is consistent with a potential involvement in tissue-specific regulation [42]. Hormone- and stress-responsive elements such as WBOXATNPR1 and ACGTATERD1 have also been associated with salicylic acid and dehydration responses [43,44]. Moreover, the frequent occurrence of light-responsive motifs (GT1CONSENSUS and GATABOX) suggests a potential association with light-responsive regulation [45]. Therefore, the abundance of these specific regulatory elements supports the hypothesis that the expression of hazelnut CDP allergens may be influenced by complex developmental cues and environmental stimuli [18]. Indeed, it has been observed that SSPs may remain silent until late embryogenesis or desiccation stages [35].

The mass spectrometry analysis of commercial hazelnut samples provided proteomic evidence for several predicted CDP allergen candidates, including peptides previously mapped to an immunoreactive 20.7 kDa polypeptide [19]. It also confirmed the presence of known Cor a allergens.

From a breeding perspective, the prioritized CDPs identified here provide potential molecular targets for reducing hazelnut allergenic potential. Although natural variation among hazelnut cultivars has been investigated, no clearly low allergenic genotype has yet been identified [46], supporting the exploration of targeted biotechnological approaches. CRISPR-Cas-based strategies could be used to target major allergen genes or simultaneously edit highly similar isoallergens and paralogs in hazelnut, including Cor a loci [10,28]. Precise editing of specific epitopic sequences, rather than complete silencing of seed storage protein genes, has also been proposed as a strategy to reduce allergenic potential while limiting the risk of pleiotropic effects [27]. The novel CDP candidates identified in this study, particularly those supported by proteomic evidence, expand the set of potential targets for future editing strategies. However, their allergenic relevance will require functional validation. Moreover, although CRISPR/Cas9-mediated editing has recently been demonstrated in hazelnut protoplasts, efficient whole-plant regeneration remains a major bottleneck for the development of genome-edited cultivars [47].

## 4. Materials and Methods

### 4.1. Dataset and CDP-Encoding Genes Annotation

To investigate the CDP superfamily evolution in hazelnut, we used the *C. avellana* cv. ‘Tombul’ genomic data [8]. The final chromosome assembly (accession number: GCA_901000735), gene model transcript, CDS and amino acid sequences, and annotations for other functional elements are available through https://treegenesdb.org/, accessed on 15 July 2025 (Supplementary Table S1). Gene annotation and protein classification strategies were previously reported by Andolfo et al. [21,48]. To discover the hazelnut CDP-encoding genes, we used three well-characterized reference Cor a genes (AF449424: *Cor a 9*; AF441864: *Cor a 11*; ON060881: *Cor a 16*) retrieved from the WHO/IUIS Allergen Nomenclature Database (http://allergen.org, accessed on 15 July 2025) in a reiterative process of gene identification. In particular, Cor a sequences were used as queries against the hazelnut proteome using a PSI-BLAST search (E-value cutoff of 1 × 10^−10^). Moreover, to detect additional hazelnut CDP-encoding genes that have escaped the foregoing annotation, the *C. avellana* proteome was scanned with the hidden Markov model (HMM) of the Cupin_1 domain (Pfam ID: PF00190) using HMMER v3.0 [49]. The analysis was carried out using the default cutoff value for statistical confidence. The protein domain architecture of identified CDPs was annotated using InterProScan v5 [50] and Conserved Domain Search (CD-Search) [51] with default parameters. The CDPs can be classified into monocupin and bicupin groups, based on the number of Cupin_1 domains [52]. A physical map of CDP-encoding loci was generated using TBtools-II software (v2.326) [53]. Gene positional data (chromosome ID, start position, end position) were extracted from *C. avellana* cv. ‘Tombul’ annotation files.

### 4.2. Multi-Sequence Alignment and Phylogenetic Analysis

MAFFT (Multiple Alignment using Fast Fourier Transform) v6.814b was used to align the Cupin_1 (Pfam ID: PF00190) domain sequences of both *C. avellana* and *A. thaliana* CDPs using the L-INS-i algorithm [54]. Evolutionary analyses were conducted using MEGA 12 [55]. The phylogenetic relationships of hazelnut CDPs were inferred using the maximum likelihood method based on the JTT model [56]. The model with the lowest BIC score (Bayesian Information Criterion) was considered to describe the substitution pattern. The bootstrap consensus tree inferred from 100 replicates was taken to represent the evolutionary history of the analyzed sequences. The trees were drawn to scale, with branch lengths measured in the number of substitutions per site. For comparative purposes, we also used 42 CDP sequences (Supplementary Table S2) from *Arabidopsis thaliana* [16]. The final phylogenetic tree was visualized using FigTree (v1.4.4) (http://tree.bio.ed.ac.uk/software/figtree/, accessed on 15 July 2025). To identify duplicated CDP-encoding gene pairs (Supplementary Figure S1), we defined a gene duplication according to the criteria reported by Gu et al. [57] and Andolfo et al. [58]. Evolutionary analyses were conducted using MEGA 12 [55].

### 4.3. Allergen Prediction and Identification of Linear and Conformational Epitopes

To discover novel allergen candidates in the hazelnut CDP superfamily, we used the web tool AllergyPred through https://allergypred.charite.de/AllergyPred [13] URL (accessed on 15 July 2025). The structural analysis of linear and conformational epitopes was carried out using two online programs, AlgPred 2.0 (https://pypi.org/project/algpred2/) [11] URL (accessed on 10 July 2025), and AllerCatPro 2.0 (https://allercatpro.bii.a-star.edu.sg) [12] URL (accessed on 12 July 2025). All predictors were used with default settings.

### 4.4. Plant Material, RNA Extraction, cDNA Synthesis, and qRT-PCR

To identify the tissue in which transcriptional changes in three hazelnut allergens (*Cor a 9*, *Cor a 11*, and *Cor a 16*) have occurred, qRT-PCR analysis was performed on immature reproductive tissues. The *C. avellana* cv. Mortarella was used in this study. Plants were grown in Nola (Naples, Italy) at the CO.N.C.I (Italian Campania Hazelnut Consortium) farm under standard agronomic practices for the area. Three biological replicates of young leaf, bracts, unripe fruit (pericarp and cotyledons), pericarp, and cotyledon samples of Mortarella were collected, approximately 40 days before harvest (immature stage), to identify the tissues in which transcriptional changes in three hazelnut allergens (*Cor a 9*, *Cor a 11*, and *Cor a 16*) have occurred. The development stage was selected based on the literature [39]. Samples were immediately frozen in liquid nitrogen at the time of collection and then stored at −80 °C. Total RNA was isolated from 100 mg of ground plant tissues using the protocol of Japelaghi et al. [59]. Quantity and quality of the isolated RNA were assessed using the NanoDrop ND-1000 spectrophotometer (Thermo Scientific, Wilmington, DE, USA) and Qubit 2.0 fluorometer (Life Technologies, Carlsbad, CA, USA). For cDNA synthesis, 100 ng of each RNA sample was reverse-transcribed using the SuperScript^®^ III cDNA Synthesis Kit (Invitrogen, Carlsbad, CA, USA) following the manufacturer’s protocol. Before use in Real-Time PCR experiments, cDNA reactions were diluted fivefold to 200 μL with UltraPure DNase/RNase-Free Distilled Water (Lonza Group, Basel, Switzerland). The expression study was conducted by real-time PCR analysis using the SYBR Green method on a 7900HT Fast Real-Time PCR System (Applied Biosystems, Foster City, CA, USA). Each 15 μL PCR reaction contained 330 nM of each primer, 2 μL of diluted cDNA, and 7.5 μL of Sybr Green Mix (Applied Biosystems). The 2−ΔΔCT method was used as a relative quantification strategy for quantitative real-time PCR (qPCR) data analysis, according to Livak and Schmittgen [60]. Young leaf tissue was used as the calibrator for ΔΔCT calculation. Amplification efficiencies of the primer pairs were comparable, as reported in Supplementary Table S3. Primer3 was used to design primers for three selected genes, *Cor a 9*, *Cor a 11*, and *Cor a 16*, and the primer for the actin gene (housekeeping) (Supplementary Table S3). To selectively amplify cDNA, primers for the housekeeping gene Chondramide A Actin (ChaActin) were designed using a portion of the ChaActin mRNA sequence (Supplementary Table S3), and ChaActin was used as the endogenous reference gene [61]. All PCR experiments were carried out in three technical replicates. Finally, to verify any contamination within the reaction mix, negative controls containing the qPCR reaction mix and molecular-grade water instead of cDNA were included.

### 4.5. Mass Spectrometry Analysis

The commercial hazelnut flour, previously characterized, was used for mass spectrometry analyses [62]. Tryptic protein digests were prepared as described by Pilolli et al. [62]. Peptide mixtures were analyzed using an Ultimate 3000 UHPLC system coupled to a Q-Exactive Plus Orbitrap mass spectrometer (Thermo Fisher Scientific, San José, CA, USA) equipped with an electrospray ionization (ESI) source. Chromatographic separation was performed on an Acclaim PepMap100, C18 column (3 μm, 100 Å, 1 × 150 mm) maintained at 25 °C. A linear gradient from 5% to 90% solvent B (acetonitrile with 0.1% formic acid) was applied at a flow rate of 250 µL/min. The operational experimental conditions for the mass spectrometer were maintained as described by Pilolli et al. [62], ensuring consistent ionization and mass-to-charge separation throughout the analysis. Raw mass spectrometry data were processed and analyzed using PEAKS Studio software (v. 12, Bioinformatics Solutions Inc., Waterloo, ON, Canada). The analysis of the mass spectra was performed using a customised protein database, including the *C. avellana* cv. ‘Tombul’ proteome and all sequences available for *C. avellana* in UniProt (https://www.uniprot.org/, accessed on 15 July 2025). Redundant protein sequences were removed using dbtoolkit-4.2.5 [63], and a custom database including 34,596 proteins was generated. The database-searching parameters were: (i) mass tolerance value of 10 ppm for the precursor ion and 0.02 Da for MS/MS fragments; (ii) enzyme: trypsin, maximum of 2 missed cleavages; (iii) fixed modification: carbamidomethyl-cysteine; variable modifications: oxidation of the methionine and deamidation (NQ). The false discovery rate was set at 1%, and to improve the confidence of protein identification, only proteins with −10lgP value > 50 were retained.

### 4.6. Detection and Distribution of CREs in the 5′ Region in Corylus

For each of the 114 CDP-encoding genes, the putative promoter region (PPR) was defined as the 2 kb genomic sequence upstream of the predicted transcription start site (TSS), reported in gene annotation. PPRs (Supplementary Table S4) were scanned on both strands to identify CREs present in the Plant Cis-acting Regulatory DNA Elements (PLACE) database [64]. CREs were clustered into six functional categories (MA: metabolic activity; TA: transcription activity; TSA: tissue-specific activity; HR: hormonal response; BSR: biotic stress response; and ASR: abiotic stress response) as previously reported by Andolfo et al. [22]. To visualize common and unique CREs among *Cor a 9*, *Cor a 11*, and *Cor a 16* allergens and new candidate allergens (Supplementary Table S5), we used TBtools software (Supplementary Figure S2) [65].

## 5. Conclusions

In this study, we identified 114 hazelnut CDPs that were classified into 12 phylogenetic clades and annotated in monocupins and bicupins. Tandem duplication events, particularly on chromosomes 1 and 2, contributed to the expansion of this superfamily. The qRT-PCR analysis showed generally low relative transcript levels of Cor a genes in immature reproductive tissues compared with young leaves, while CRE prediction in CDP PPRs identified abundant hormone-responsive elements. Finally, mass spectrometry analysis detected seven of the eight prioritized CDPs, together with Cav10g09900, which did not meet the combined computational cut-offs. Overall, these findings advance our understanding of the molecular evolution of hazelnut CDP-encoding genes.

## Figures and Tables

**Table 1:** Allergen gene candidates predicted in the *C. avellana* cv. ‘Tombul’ genome.

Gene Locus ID *		AllergyPred	AlgPred v2	AllerCatPro v2
		(Allergenic Probability (0–1)	Hybrid Score (0–2)	3D Epitope Identity (%)
Cav02g11550	(*Cor a 11*)	0.98	1	100.0
Cav04g12115	(*Cor a 16*)	0.98	1.66	92.9
Cav04g14050		0.98	1.65	86.7
Cav04g14080		0.98	1.65	86.7
Cav11g01160	(*Cor a 9*)	0.98	1.95	100.0
Cav11g01180	(*Cor a 9* isoform)	0.98	1.98	100.0
Cav11g01190	(*Cor a 9* isoform)	0.98	1.86	100.0
Cav11g15560		0.98	1.52	100.0

* Cor a gene annotation was reported by Amoroso and Andolfo [10].

**Table 2:** Number of CRE occurrences in the PPRs of novel allergen candidates.

Functional Categories *	Normalized Number of Identified CREs				
	Cav04g14050	Cav04g14080	Cav11g01180	Cav11g01190	Cav11g15560
TA ^b^	207	231	224	217	230
ASR ^b^	207	247	123	184	184
MA ^e^	116	138	140	158	138
HR ^d^	80	78	124	73	118
TSA ^a^	263	268	270	320	275
BSR ^b^	195	176	200	200	238
Total	1068	1137	1082	1152	1183

Normalized value refers to the abundance of each CRE class in the total CREs belonging to the same class. * Data were analyzed by two-way analysis of variance following Duncan’s test, and different letters represent a statistical difference (*p* < 0.05).

**Table 3:** Hazelnut CDPs identified via HPLC-ESI-MS/MS analysis. Statistical significance, protein coverage, molecular weight (MW) and number of corresponding peptides identified in the hazelnut kernel were reported for each well-known and new potential CDP allergens. The de novo sequenced peptides mapped to the immunoreactive 20.7 kDa polypeptide by Nitride et al. [19] were indicated. Full unique peptide sequence data and confidence scores are provided in Supplementary Table S11.

Gene Locus IDs *	−10 LgP	Protein Coverage (%)	MW (kDa)	N. of Mapped Peptides		De Novo Sequenced Peptides
				Multi-Match	Unique-Match	(Nitride et al., 2013 [19])
Cav02g11550 (*Cor a 11*)	169	62	53.3	37	2	
Cav04g12115 (*Cor a 16*)	185	40	13.8	53	53	
Cav04g14050	184	77	53.4	54	53	YLQLSAER, GVEETFCTLR, and AIPEDVLINSYR
Cav04g14080						
Cav11g01160 (*Cor a 9*)	204	77	59.0	87	2	AGSQGFEWVSFK, TNDNAQISQLAGR, HDGQNLFDWLR and ITSLNSLNLPILR
Cav11g01180 (*Cor a 9* isoform)	205	81	58.8	92	15	
Cav11g01190 (*Cor a 9* isoform)	197	75	55.9	77	53	
Cav10g09900	150	42	57.5	22	22	

* Cor a gene annotation was reported by Amoroso and Andolfo [10].

## Data Availability

All the data are shared in the Supplementary Materials available online. Mass spectrometry data will be made available by the R.P. and C.N. upon request.

## Supplementary Materials

The following supporting information can be downloaded at: https://www.mdpi.com/article/10.3390/ijms27198924/s1.

## Abbreviations

ASR	abiotic stress response
BSR	biotic stress response
CDPs	Cupin_1 domain-containing proteins
Cor a	*Corylus avellana*
CREs	cis-acting regulatory elements
HR	hormonal response
MA	metabolic activity
PPRs	putative promoter regions
SSPs	seed storage proteins
TA	transcription activity
TSA	tissue-specific activity
